# Chitosan-Coated Gold Nanoparticles Induce Low Cytotoxicity and Low ROS Production in Primary Leucocytes, Independent of Their Proliferative Status

**DOI:** 10.3390/pharmaceutics13070942

**Published:** 2021-06-24

**Authors:** Helen Yarimet Lorenzo-Anota, Diana G. Zarate-Triviño, Jorge Alberto Uribe-Echeverría, Andrea Ávila-Ávila, José Raúl Rangel-López, Ana Carolina Martínez-Torres, Cristina Rodríguez-Padilla

**Affiliations:** 1 Facultad de Ciencias Biológicas, Laboratorio de Inmunología y Virología, Monterrey, Universidad Autónoma de Nuevo León, Nuevo León 66455, Mexico; helen.lorenzoant@uanl.edu.mx (H.Y.L.-A.); diana.zaratetr@uanl.edu.mx (D.G.Z.-T.); alberto.uribeechvr@uanl.edu.mx (J.A.U.-E.); andrea.avilaav@uanl.edu.mx (A.Á.-Á.); jose.rangellpz@uanl.edu.mx (J.R.R.-L.); cristina.rodriguezpd@uanl.edu.mx (C.R.-P.); 2 LONGEVEDEN SA de CV, Monterrey, Nuevo León 64710, Mexico

**Keywords:** gold nanoparticles, proliferation, ROS, cancer, chitosan, lymphoid cells, LPS, ConA, PBMCs

## Abstract

(1) Background: Chitosan-coated gold nanoparticles (CH-AuNPs) have important theranostic applications in biomedical sciences, including cancer research. However, although cell cytotoxicity has been studied in cancerous cells, little is known about their effect in proliferating primary leukocytes. Here, we assessed the effect of CH-AuNPs and the implication of ROS on non-cancerous endothelial and fibroblast cell lines and in proliferative lymphoid cells. (2) Methods: The Turkevich method was used to synthetize gold nanoparticles. We tested cell viability, cell death, ROS production, and cell cycle in primary lymphoid cells, compared with non-cancer and cancer cell lines. Concanavalin A (ConA) or lipopolysaccharide (LPS) were used to induce proliferation on lymphoid cells. (3) Results: CH-AuNPs presented high cytotoxicity and ROS production against cancer cells compared to non-cancer cells; they also induced a different pattern of ROS production in peripheral blood mononuclear cells (PBMCs). No significant cell-death difference was found in PBMCs, splenic mononuclear cells, and bone marrow cells (BMC) with or without a proliferative stimuli. (4) Conclusions: Taken together, our results highlight the selectivity of CH-AuNPs to cancer cells, discarding a consistent cytotoxicity upon proliferative cells including endothelial, fibroblast, and lymphoid cells, and suggest their application in cancer treatment without affecting immune cells.

## 1. Introduction

Currently, cancer is still the main cause of death for patients worldwide, with increasing incidence [1]. Cancer cells are characterized by uncontrolled division and proliferation, and by their ability to invade other tissues [2]. It is currently accepted that the proliferative signaling pathways in cancer cells harbor one or more driving alterations that provide them a survival edge [3,4]. Therefore, their cell-death resistance and the continuous replicative state of cancer cells limits the success of current treatments [3,4,5]. Additionally, most cancer treatments promote immunosuppression, as they are highly cytotoxic to proliferating non-cancer cells, which is the case of immune-system cells.

The application of nanotechnology in medicine seeks to innovate with new techniques and materials for diagnosis, treatment, and prevention therapies for different diseases [6,7]. Given their nanometric size, nanoparticles (NPs) are considered a possible treatment for cancer, as they can accumulate in tumor tissues (potential improvement of the therapeutic effect), show a reduced systemic toxicity [8], and their surface has the capacity to be functionalized, which can lead to a targeted therapy [9,10]. Gold nanoparticles (AuNPs) have been broadly studied, benefiting from their unique chemical, electrical, and optical properties and excellent biocompatible features, as well as the ease of synthetic manipulation and precise control over their physicochemical properties [11,12]. The use of gold nanoparticles (AuNPs) is growing rapidly, and nowadays there is an increasing range of applications for their use [13,14]. It has been observed that they have an important biological potential, as the characteristics of the NPs’ surface provide high biocompatibility [15,16].

In previous reports, we demonstrated the cytotoxic effect and characterized the cell death mechanism of chitosan-coated gold nanoparticles (CH-AuNPs) in tumor (HeLa and MCF-7) [17] and leukemic (CEM and K562) cell lines [18]. CH-AuNPs cytotoxicity is ROS-dependent in all cancerous cells and is independent of the cell lineage, interestingly without being cytotoxic to primary lymphoid cells (peripheral blood mononuclear cells (PBMCs) and bone marrow cells (BMCs)). However, it is important to determine the effect of CH-AuNPs on non-cancer cells and on lymphoid cells even during proliferative stimuli to determine if the proliferative condition, for example, during an infectious process, defines the selectivity.

Thus, the purpose of this study was to determine the cytotoxicity of CH-AuNPs on endothelial, fibroblast, and lymphoid cells, and to evaluate whether the proliferative status of lymphoid cells will favor CH-AuNPs’ cytotoxicity. We tested cell viability by MTT and by flow cytometry, and we analyzed cell death (Ann-V and PI), cell cycle (PI), and ROS production (DCFDA and DHE) in cancer and non-cancer cell lines, and in primary lymphoid cells. We also evaluated the cytotoxic effect of CH-AuNPs in lymphoid cells with or without the mitogens concanavalin A (ConA) and lipopolysaccharide (LPS), when compared with the chemotherapy etoposide and sodium citrate-AuNPs (SC-AuNPs).

## 2. Materials and Methods

### 2.1. Cell Culture

Non-small-cell lung cancer cells A549 (ATCC^®^ CCL-185™), human umbilical vascular endothelial cells HUVEC (ATCC^®^ CRL-1730™), murine embryonic fibroblasts NIH3T3 (ATCC^®^ CRL-1658™), human T-acute lymphoblastic leukemia cells Jurkat Clone E6-1 (ATCC^®^ TIB-152™), and CEM (ATCC^®^ CCL-119™), and murine lymphoma cells L5178-R (ATCC^®^ CRL-1722™) were obtained from the American Type Culture Collection (ATCC, Manassas, VA, USA) and maintained under suggested conditions. A549, HUVEC, and NIH3T3 were cultured in plastic sterile flasks (Life Technologies, Grand Island, NY, USA) at 37 °C in 5% CO_2_ atmosphere, using DMEM F-12 medium (Life Technologies, Grand Island, NY, USA) supplemented with 1 µg/mL amphotericin B, 1 µg/mL penicillin and 2.5 × 10^−3^ µg/mL streptomycin, and 10% FBS (Life Technologies, Grand Island, NY). Jurkat, CEM, and L5178Y-R were cultured in plastic sterile flasks (Life Technologies, Grand Island, NY) at 37 °C in 5% CO_2_ atmosphere, using RPMI 1640 medium (Life Technologies, Grand Island, NY) supplemented with 1 µg/mL amphotericin B, 1 µg/mL penicillin and 2.5 × 10^−3^ µg/mL streptomycin, and 10% FBS (Life Technologies, Grand Island, NY, USA).

This study was approved by the Institutional Ethics Committee at the Universidad Autónoma de Nuevo León, College of Biological Sciences. After obtaining written informed consent, peripheral blood mononuclear cells (PBMCs) were obtained from healthy donors. PBMCs were isolated by density-gradient centrifugation with Ficoll-Paque™ PLUS (GE Healthcare, Chicago, IL, USA) and maintained at 5 × 10^6^ cells/mL in cell-culture plates at 37 °C in 5% CO_2_ atmosphere, using RPMI 1640 medium (Life Technologies, Grand Island, NY, USA) supplemented with 1 µg/mL amphotericin B, 1 µg/mL penicillin and 2.5 × 10^−3^ µg/mL streptomycin, and 10% FBS (Life Technologies, Grand Island, NY, USA).

The Animal Ethical Committee (CEIBA) approved and accepted the use of animals for this study (Number: 01/2015). The experiments were conducted according to Mexican regulation NOM-062-ZOO-1999. After ethical sacrifice, bone marrow cells (BMCs) were obtained from only one femur and tibia per healthy mouse (male, 6–8 weeks). Splenic mononuclear cells were obtained from spleen by perfusion and isolated by density-gradient centrifugation with Ficoll-Paque™ PLUS (GE Healthcare, Chicago, IL, USA) of healthy mouse (male, 6–8 weeks). BMCs and splenic mononuclear cells were maintained at 5 × 10^6^ cells/mL at 37 °C in 5% CO_2_ atmosphere, using RPMI 1640 medium (Life Technologies, Grand Island, NY) supplemented with 1 µg/mL amphotericin B, 1 µg/mL penicillin and 2.5 × 10^−3^ µg/mL streptomycin, and 10% FBS (Life Technologies, Grand Island, NY, USA).

### 2.2. Nanoparticle Synthesis and Characterization

#### 2.2.1. Nanoparticle Synthesis

The Turkevich method, previously described in [19], was used to synthetize the CH-AuNPs and SC-AuNPs. For the CH-AuNP synthesis, we prepared an acid solution of chitosan (CH, 2% *w/w* in acetic acid 0.4 M) by dissolving CH (medium molecular weight, 300,000 g/mol, with 75–85% of deacetylation) in 2 mM hydrochloroauric acid solution (HAuCl_4_), then we homogenized the solution on a magnetic plate at room temperature for 15 min at 80–90 rpm until it changed to the color of red wine [18]. For the SC-AuNP synthesis, sodium citrate and HAuCl_4_ were purchased from Sigma-Aldrich, sodium citrate was dissolved in distilled water to obtain a 1% solution, the 1 mM HAuCl_4_ solution was mixed with sodium citrate and placed in a water bath for 15 min at 100 °C ± 2 °C, to a ratio of 1:1 (HAuCl_4_/sodium citrate) volume/volume, until it changed to the color of red wine. Finally, the synthesis was allowed to settle at room temperature, and was stored for later use. The CH-AuNPs and SC-AuNPs were diluted 1:1 in RPMI 1640 medium (GIBCO^®^ by Life Technologies). The concentrations were determined based on precursor salt (HACl_4_) concentration (μM) involved in AuNPs synthesis.

#### 2.2.2. Nanoparticle Characterization

Ultraviolet-visible spectroscopy was used to determine the surface plasmon resonance using a NanoDrop 2000c spectrophotometer (Thermo Fisher Scientific, Bartlesville, OK, USA). Dynamic light scattering (DLS) in a Zetasizer ZS90-Nano (Malvern Instruments, Malvern, United Kingdom) was implemented to determine zeta potential (ZP). Mean particle diameter was measured by dynamic light scattering (DLS) using a Nanosizer NS90 (Siemens, Malvern, PA, USA). For analysis, samples were diluted in distilled water (1:1000). Transmission electron microscopy (TEM) in a field-emission gun (FEI TITAN G2 80-300) operated at 300 kV was employed to confirm the size of the AuNPs.

### 2.3. Cell-Viability Assay

Relative cell viability was determined by MTT; this assay consisted of measuring and quantifying spectrophotometric means of yellow tetrazolium (3-(4,5-dimethylthiazilyl-1)-2,5-diphenyl tetrazolium bromide) (Milliporesigma) reduction by metabolic activity of the cells to purple formazan. In 96-well microtiter plates (Corning), 5 × 10^3^ cells per well were seeded. Cells were treated with CH-AuNPs, chitosan, HACl_4_, or SC-AuNPs, at different concentrations (25, 50, 75, 100, and 125 μM) for 24 h. Based on the μM concentration of the precursor salt (HACl_4_), the concentrations of CH-AuNPs and SC-AuNPs (μM) were used for the synthesis of AuNPs. After treatment, PBMCs were centrifuged at 400× *g* for 10 min and carefully decanted, then MTT solution (2 mg/mL in phosphate-buffered saline (PBS)) was added to each well and incubated for two hours at 37 °C. Finally, MTT solution in the medium was aspirated, and cells were dissolved with dimethyl sulfoxide (DMSO) (Milliporesigma, Eugene, OR, USA) to solubilize the formazan crystals formed in the viable cells. The optical density was measured at 570 nm using a microplate reader (Synergy2, Biotek, Winooski, VT, USA).

### 2.4. Cell-Death Analysis

Cell death was determined by analyzing phosphatidylserine exposure and cell-membrane permeabilization, using Annexin V-allophycocyanin (APC) (AnnV, 0.25 µg/mL; BD Biosciences Pharmingen, San Jose, CA, USA) and propidium iodide (PI; 0.5 μg/mL; Milliporesigma, Eugene, OR, USA), respectively, after 24 h of CH-AuNP treatment. In brief, 5 × 10^4^ cells per well in 24-well plates (Corning Inc. Costar^®^, Corning, NY, USA) were seeded and treated with different concentrations of CH-AuNPs (25, 50, 75, 100, and 125 μM); this allowed us to define the median cytotoxic concentration (CC_50_) of CH-AuNPs required to reduce cell viability by 50% (CC_25_, CC_50_, and CC_75_). After treatment, cells were washed and resuspended in binding buffer (10 mM HEPES/NaOH pH 7.4, 140 mM NaCl, 2.5 mM CaCl_2_), and stained with AnnV (0.1 μg/mL) and PI (0.5 μg/mL) for 30 min at 4 °C. Cells were analyzed by flow cytometry (fluorescence-activated cell sorting (FACS); BDAccury6; Becton Dickinson, San Jose, CA, USA) using FlowJo Software (Tree Star Inc., Ashland, OR, USA).

### 2.5. Reactive Oxygen Species (ROS) Analysis

ROS production levels were measured using two staining methods, dihydroethidium (DHE; Invitrogen, St Louis, MO, USA) for O_2_^−^ quantification and dichlorodihydrofluorescein diacetate (DCFDA; Invitrogen, St Louis, MO, USA) to quantify H_2_O_2_ levels by flow cytometry. In brief, 5 × 10^4^ cells per well were seeded in 24-well plates (Corning Inc. Costar^®^, Corning, NY, USA) and treated with CC_50_ of CH-AuNPs for 24 h. After treatment, cells were washed and stained with DHE (1 μM) or DCFDA (0.25 μM) and incubated for 30 min at 37 °C. The analysis was done by flow cytometry using FlowJo Software (Tree Star Inc., Ashland, OR, USA).

### 2.6. ROS Inhibitor

N-acetyl-cysteine (NAC; 5 mM; Sigma-Aldrich, St Louis, MO, USA), a ROS inhibitor, was used to determine ROS implication in the cell-death mechanism. NAC was added 30 min before CH-AuNP treatment [20].

### 2.7. Cell Cycle Analysis

Cell cycle analysis was evaluated trough intracellular DNA quantification, using propidium iodide (PI) staining by flow cytometry. In 6-well dishes, 5 × 10^5^ cells were seeded and treated with CC_25_, CC_50_, and CC_75_ of CH-AuNPs for 24 h. Later, we washed and fixed with 70% ethanol overnight. After fixation, cells were washed again and incubated with PI (10 μg/mL; Milliporesigma) and simultaneous RNase (Sigma-Aldrich, USA) for 30 min at 37 °C. DNA degradation and cell DNA contents for the cell cycle were measured by flow cytometry and analyzed in FlowJo Software (Tree Star Inc., Ashland, OR, USA). A SubG1 population analysis was used for DNA degradation quantification.

### 2.8. Proliferative Analysis

For the assessment of PBMC proliferation, we used carboxyfluorescein succinimidyl ester (CFSE; 10 mM; Invitrogen, Carlsbad, CA, USA), which was added to the PBMC suspension. Samples were vortexed and incubated at 37 °C in the dark for 10 min. PBMCs were washed twice with warm PBS. PBMCs were incubated with mitogen concanavalin A (ConA; 5 μg/mL; Sigma-Aldrich, Darmstadt, Germany) for 96 h. PBMCs with ConA were maintained at 4 × 10^6^ cells/mL in cell-culture plates at 37 °C in 5% CO_2_ atmosphere, using RPMI 1640 medium (GIBCO^®^ by Life Technologies) supplemented with 1 µg/mL amphotericin B, 1 µg/mL penicillin and 2.5 × 10^−3^ µg/mL streptomycin (GIBCO^®^ by Life Technologies), and 10% FBS (GIBCO^®^ by Life Technologies).

### 2.9. Proliferative Stimuli

To induce proliferation in primary cell cultures (BMCs, PBMCs, and splenic mononuclear cells) we treated with lipopolysaccharide (LPS; 10 μg/mL) or concanavalin A (ConA; 5 μg/mL) during CH-AuNP treatment for 24 h.

### 2.10. Statistical Analysis

The data were analyzed using GraphPad Prism (GraphPad Software, San Diego, CA, USA). The results given in this study represent the mean of at least three independent experiments done in triplicate (mean ± SD). Statistical analysis was done using a paired Student’s *t*-test. The statistical significance was defined as *p* < 0.05.

## 3. Results

### 3.1. Gold Nanoparticles

**Figure 1:**
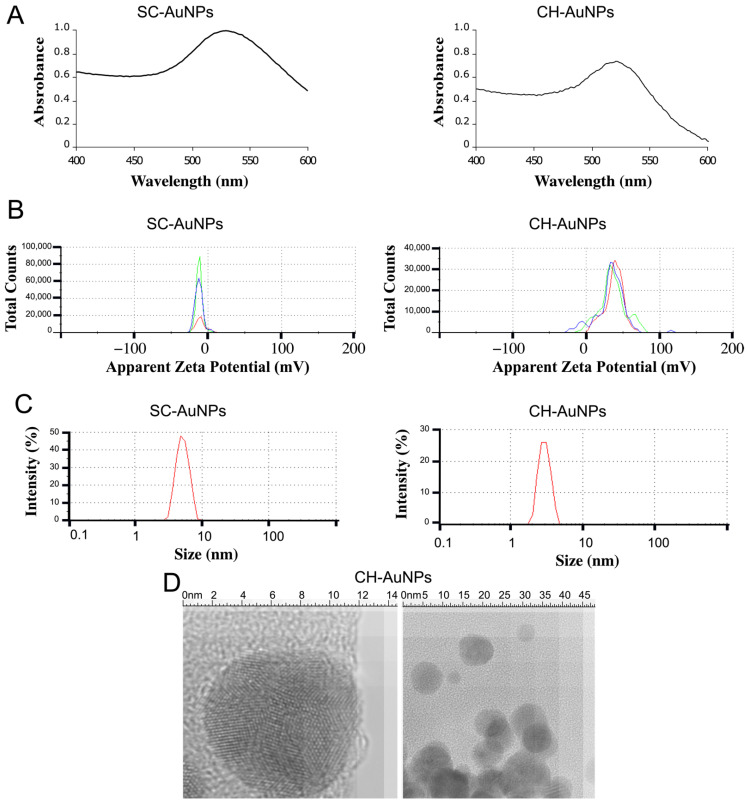
Structure and characterization of SC-AuNPs and CH-AuNPs. (**A**) UV-VIS surface plasmon spectrum of SC-AuNPs and CH-AuNPs. (**B**) Surface charge measured by zeta-potential analysis of SC-AuNPs and CH-AuNPs. (**C**) Size distribution obtained from dynamic light scattering (DLS) analysis of SC-AuNPs and CH-AuNPs. (**D**) Size of CH-AuNPs determined by transmission electron microscopy (TEM).

SC-AuNPs and CH-AuNPs showed a typical surface plasmon resonance of around 520 nm (Figure 1A). SC-AuNPs revealed a zeta potential (ZP) of −10 mV, and CH-AuNPs exhibited a positive ZP of +36.7 mV (Figure 1B). The average size was tested by dynamic light scattering (DLS), and SC-AuNPs revealed a size of 3–10 nm, with a mean value of 3.5 nm, while CH-AuNPs showed an average size of 3–10 nm, with a mean value of 3.75 nm (Figure 1C); in both cases, the polydispersity was 0.3. We corroborated the size and shape of the CH-AuNPs by transmission electron microscopy (TEM), and the average size was 7.78 nm (Figure 1D), which corresponded to the average size detected by DLS.

### 3.2. CH-AuNPs Induce Low Affections in Endothelial, Fibroblast, and Peripheral Blood Mononuclear Cells, and High Selective Cytotoxicity in Cancer Cell Lines

**Figure 2:**
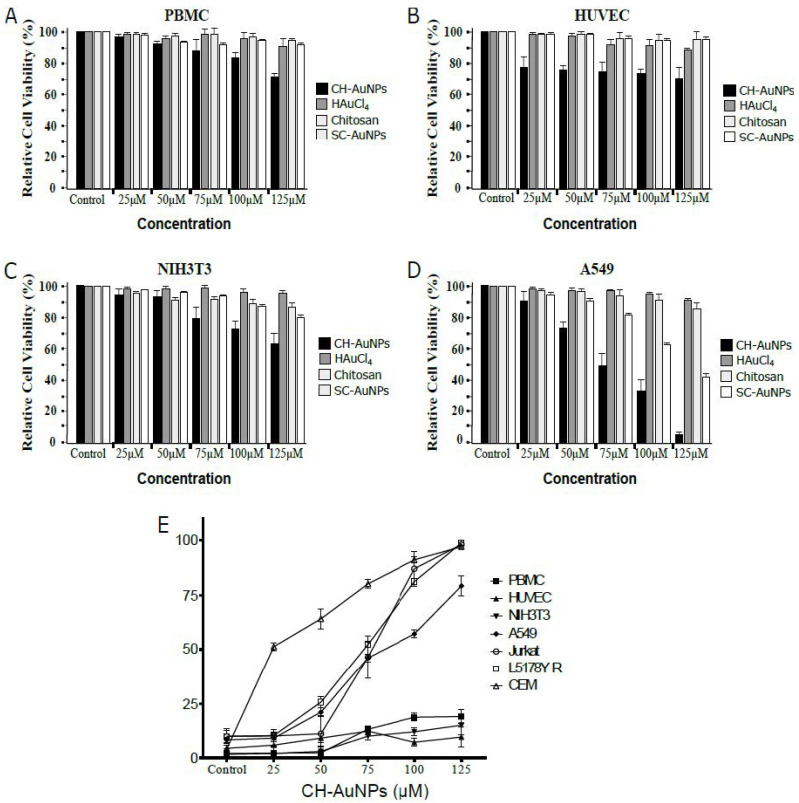
Cytotoxic effect of CH-AuNPs, HAuCl_4_, chitosan, and SC-AuNPs in non-cancer and cancer cells. (**A**) PBMCs were treated with different concentrations of CH-AuNPs, HAuCl_4_, chitosan, and SC-AuNPs (25, 50, 75, 100, and 125 μM) for 24 h. (**B**) HUVEC, (**C**) NIH3T3, and (**D**) A549 cells were treated as in (**A**). Cell viability was measured by MTT assay. The percentages refer to relative cell viability represented as percentage of control (non-treated cell viability = 100%). (**E**) Quantification of cell death by flow cytometry using annexin V (phosphatidylserine exposure analysis) and propidium iodide (membrane-permeability analysis) staining in PBMC, HUVEC, NIH3T3, A549, Jurkat, L5178Y-R, and CEM cells treated with different concentrations (25, 50, 75, 100, and 125 μM) of CH-AuNPs for 24 h. The results are presented as mean ± standard deviation of three different experiments.

We tested cell viability after treatment with CH-AuNPs, synthesis precursors (Chitosan and HAuCl_4_), and SC-AuNPs (used as AuNP control) by MTT assay in peripheral blood mononuclear cells (PBMCs), human umbilical vascular endothelial cells (HUVECs), murine embryonic fibroblast cells (NIH3T3s), and the non-small-cell lung cancer cell line A549. Cell viability of PBMCs (Figure 2A) was only slightly decreased (less than 30%) at 100 μM of CH-AuNP treatment, similar to HUVECs (Figure 2B) and NIH3T3s (Figure 2C). Furthermore, neither chitosan, HAuCl_4_ alone, nor SC-AuNPs were cytotoxic. In contrast, A549 cells showed a concentration-dependent loss of cell viability, with a mean inhibitory concentration (IC_50_) of 75 μM and a complete inhibitory concentration (IC_100_) of 125 μM of CH-AuNP treatment (Figure 2D). To correlate the loss of cell viability with CH-AuNP cytotoxicity and discard a metabolic alteration, we assessed cell death by analyzing phosphatidylserine exposure and membrane permeability. Cell-death analysis of non-cancer cells (PBMCs, HUVECs, and NIH3T3s) versus cancer cells (human tumor cell line A549, human leukemic cell line Jurkat, and mouse lymphoblast cell line L5178Y-R) is visualized in Figure 2E. We used the CEM cell line as a positive control, as it was previously reported to be sensitive to CH-AuNPs [18]. Human PBMCs, HUVECs, and NIH3T3s did not present more than 20% of cell death at 125 μM of CH-AuNPs. However, A549 cells presented 25% of cell death (CC_25_) at 25 μM, increased in a dose-dependent manner, with mean cytotoxic concentration (CC_50_) at 75 μM, and CC_75_ at 125 μM, after 24 h of CH-AuNP treatment. Compared to CEM cells, Jurkat and L5178Y-R cells were more resistant to CH-AuNP treatment, and showed a CC_50_ at 50 μM and CC_100_ at 100 μM of CH-AuNPs. This confirmed that the CH-AuNPs were selective cell-death inductors only in cancer cells, and not their synthesis precursors, and revealed cancer cells’ susceptibility. Taken together, these results indicated that CH-AuNPs did not significatively affect the integrity of non-malignant cells, regardless of the species (murine or human).

### 3.3. CH-AuNPs Induce Different ROS Profiles in Cancer and Non-Cancer Cells

**Figure 3:**
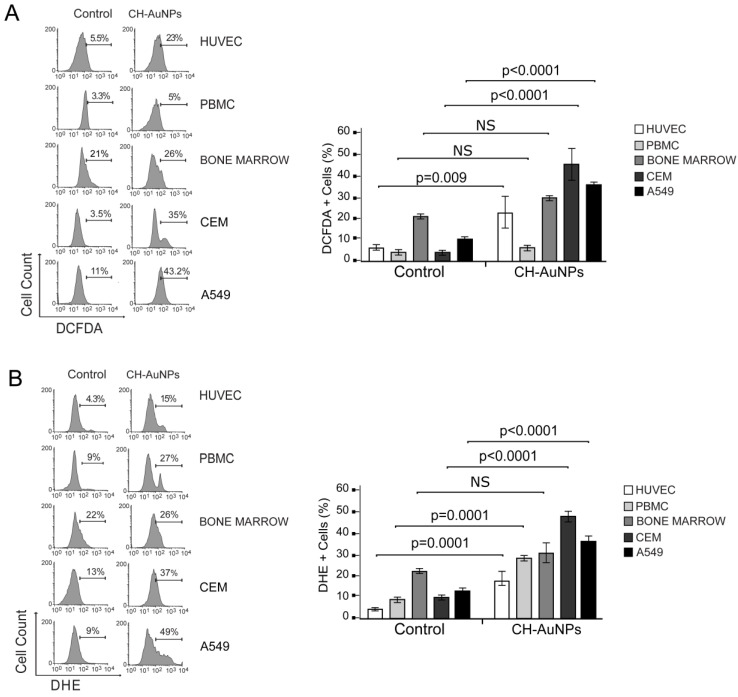
ROS production analysis in cancer and non-cancer cells upon CH-AuNP treatment. (**A**) Analysis (left) and quantification (right) of ROS (H_2_O_2_) production by flow cytometry using DCFDA staining in non-cancer cells (HUVECs, PBMCs, and BMCs) and cancer cell lines (CEM and A549) treated with CH-AuNPs for 24 h. (**B**) Analysis (left) and quantification (right) of ROS (O_2_^−^) production using DHE staining by flow cytometry in non-cancer cells (HUVEC cells, PBMC and BMC) and cancer cell lines (CEM and A549) upon CH-AuNP treatment for 24 h. The results are presented as mean ± standard deviation of three different experiments. NS = not significant.

CH-AuNPs increase ROS production in cancer cells, essential in the cell-death mechanism [17,18], and could be a substantial feature in selective cytotoxicity to cancer cells. To reveal the CH-AuNPs’ effect on non-cancer cells, we tested intracellular ROS levels using two different dyes, DCFDA (which has affinity principally to H_2_O_2_) [21] and DHE (which has affinity to O_2_^−^) [22] in the HUVEC cell line, PBMCs, and lymphoid cells derived from mouse bone marrow (BM), and compared them to cancer cell lines (CEM and A549). CH-AuNPs increased DCFDA fluorescence in HUVECs (23%) compared to untreated cells (5.5%), whereas in PBMCs, we observed 5.5% fluorescence in CH-AuNPs-treated cells and 3.3% in the control; in BM cells, CH-AuNP treatment increased the fluorescence from 21% in the control to 26% in the treated cells (Figure 3A). This was in contrast to the CEM cell line, in which CH-AuNPs enhanced fluorescence from 3.5% to 35%; and in A549, from 11% to 43.3% (Figure 3A). On the other hand, the DHE analysis showed that CH-AuNPs increased the fluorescence from 4.3% to 15% in HUVECs, and from 9% to 27% in PBMCs (Figure 3B). In BM cells, DHE fluorescence was not significatively modified after CH-AuNP treatment (26%) when compared to the control (22%). In cancer cells, the fluorescence potentiated to 37% and 49% in CEM and A549 cells, respectively (Figure 3B). These results revealed that CH-AuNPs increased different ROS production depending on the cell type, being O_2_^−^ in PBMCs and H_2_O_2_ and O_2_^−^ in endothelial cells.

### 3.4. ROS Production Promoted by CH-AuNPs Is Crucial to Low Cytotoxicity in Non-Cancer Cells

**Figure 4:**
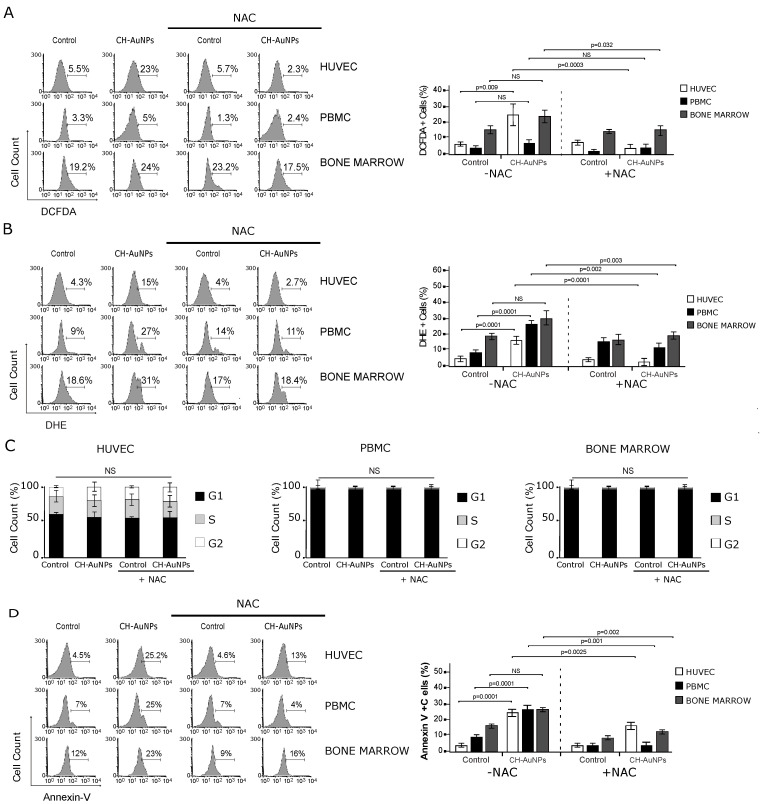
ROS production analysis and implication in cell cycle and cell death in HUVECs, PBMCs and BMCs upon CH-AuNP treatment. (**A**) Analysis (left) and quantification (right) of ROS (H_2_O_2_) production by flow cytometry using DCFDA staining and NAC as a ROS inhibitor in HUVECs, PBMCs, and BMCs treated with CH-AuNPs for 24 h. (**B**) Analysis (left) and quantification (right) of ROS (O_2_^−^) production using DHE staining and NAC as a ROS inhibitor by flow cytometry in HUVECs, PBMCs, and BMCs upon CH-AuNP treatment for 24 h. (**C**) Quantification of cell-cycle analysis in HUVECs, PBMCs, and BMCs treated with CH-AuNPs and using NAC as a ROS inhibitor for 24 h. (**D**) Analysis (left) and quantification (right) of phosphatidyl serine exposure analysis by flow cytometry using annexin V-APC (AnnexinV) staining and NAC as a ROS inhibitor in HUVECs, PBMCs, and BMCs treated with CH-AuNPs for 24 h. The results are presented as mean ± standard deviation of three different experiments. NS = not significant.

Intracellular ROS play an important role in numerous physiological (including cell-cycle progression, proliferation, and cell death) and pathological processes (cancer progression) [23,24]. While ROS promote cell death in cancer cells, [17,18] their implication in non-cancer cells is still unknown. Thus, the role of ROS in non-cancerous cells’ cytotoxicity was assessed. In Figure 4A, we show that NAC prevented DCFDA fluorescence induced by CH-AuNPs on HUVECs (2.3%) and BM cells (17.5%), but not significatively in PBMCs (2.4%). Complementary, NAC avoided the DHE-fluorescence induced by CH-AuNPs on HUVECs (2.7%), PBMCs (11%), and BM cells (18.4%) (Figure 4B). The antioxidant NAC prevented H_2_O_2_ and O_2_^−^ production induced by CH-AuNPs in non-cancer cells. It is unknown whether CH-AuNPs or ROS induced by CH-AuNPs can modify the cell cycle of non-cancer cells, so we then assessed the cell cycle in the presence and absence of NAC. CH-AuNPs did not induce cell-cycle modifications in HUVECs, PBMCs, or BM cells (Figure 4C), even during NAC treatment with respect to the control, which suggested that neither CH-AuNPs nor ROS production induced by CH-AuNPs affected the cell cycle in non-cancer cells.

Next, we assessed the role of ROS in cell death. In Figure 4D, we depict representative dot plots where NAC inhibited the low cell death induced by CH-AuNPs from 25% to 13% in HUVECs, from 25% to 4% in PBMCs, and from 23% to 16% in BM cells. This revealed that the low levels of ROS induced by CH-AuNPs in non-cancer cells were crucial for low cytotoxicity, and that ROS detected by DHE (O_2_^−^) might be implicated in cell death.

### 3.5. CH-AuNPs Induce Low O_2_^−^ Production and Low Cytotoxicity in Proliferative PBMCs

**Figure 5:**
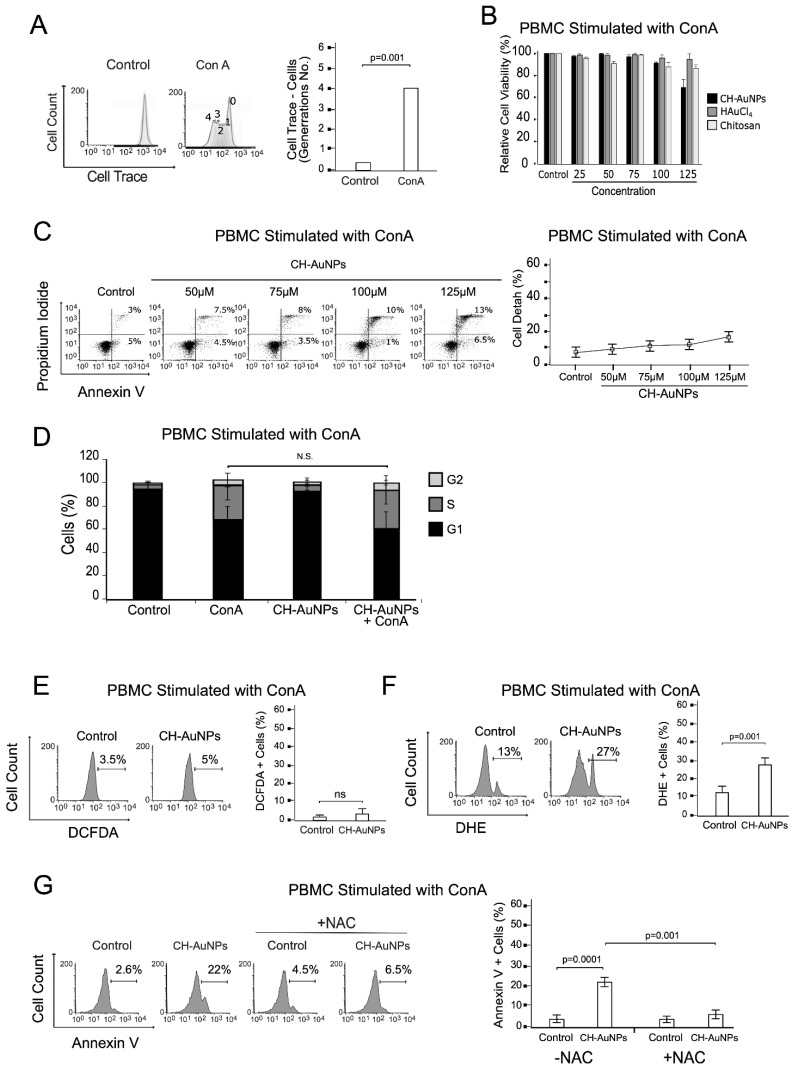
Effect of CH-AuNPs on primary PBMCs stimulated with the mitogen concanavalin A. (**A**) Representative histograms (**right**) and quantification (**left**) of cell proliferation by flow cytometry through CFSE staining in PBMCs left untreated or treated with the mitogen concanavalin A (ConA) for 96 h. (**B**) Cell-viability analysis of PBMCs with ConA treated with CH-AuNPs, HAuCl_4_, and chitosan (25, 50, 75, 100, and 125 μM) for 24 h. (**C**) Cell-death analysis (**left**) and quantification (**right**) of PBMCs with ConA treated with CH-AuNPs (25, 50, 75, 100, and 125 μM) for 24 h. (**D**) Quantification of cycle distribution of PBMCs with or without ConA and treated with 125 μM of CH-AuNPs for 24 h. (**E**) ROS (H_2_O_2_) analysis (**left**) and quantification (**right**) by flow cytometry through DCFDA staining of PBMCs with ConA treated with CH-AuNPs for 24 h. (**F**) ROS (O_2_^−^) analysis (**left**) and quantification (**right**) by flow cytometry through DHE staining of PBMCs with ConA treated with CH-AuNPs for 24 h. (**G**) Representative dot plots of cell-death analysis (**left**) and quantification (**right**) of PBMCs with ConA treated with CH-AuNPs for 24 h, using NAC as a ROS inhibitor for 24 h. The results are presented as mean ± standard deviation of three different experiments. N.S. = not significant.

Since most chemotherapies affect proliferating healthy cells, we assessed the effect of CH-AuNPs on proliferating PBMCs. PBMCs were stimulated with the mitogen concanavalin A (ConA) and then treated with CH-AuNPs for 24 h. ConA is a mitogenic lectin (polyclonal activator) that activates lymphocytes, including memory-type cells, irrespective of their antigenic specificity [25]. First, we confirmed proliferative PBMC status. Human PBMCs were labeled with CFSE before treatment with ConA, and after 96 h of mitogenic stimulation, several peaks with lower CFSE intensity were detected in the CFSE profiles, indicating that multiple rounds of cell division occurred during this time frame (Figure 5A). Once we confirmed the PBMCs’ proliferative status, the next step evaluated cell viability. Figure 5B shows the relative cell viability analysis, revealing that CH-AuNPs did not decrease cell viability, even at 125 μM, similar to their synthesis precursors chitosan and HAuCl_4_ (Figure 5B). To discard the cytotoxicity of CH-AuNPs in proliferating PBMCs, we assessed cell death. CH-AuNPs did not increase fluorescence for annexin V and PI more than 20% at 125 μM of CH-AuNPs (CC_100_ in cancer cells) (Figure 5C). Cell-cycle alterations induced by CH-AuNPs were evaluated in PBMCs stimulated with ConA. ConA increases the percentage of cells in phase S and G2, when compared to control PBMCs without ConA (Figure 5D). In addition, we did not observe cell-cycle modifications in PBMCs stimulated with ConA after CH-AuNP treatment when compared to untreated PBMCs stimulated with ConA (Figure 5D). Thus, CH-AuNPs did not affect the cell integrity or cell-cycle progression of proliferative PBMCs.

The HUVEC line and PBMCs converged on O_2_^−^ production, which was low when compared to cancer cell lines, indicating that ROS played a crucial role in cell death. Thus, we tested H_2_O_2_ and O_2_^−^ in proliferative PBMCs. We did not observe differences in fluorescence to DCFDA in untreated cells (3.5%) and treated cells (5%), indicating that treatment did not enhance H_2_O_2_ production (Figure 5E). However, in the DHE analysis, we observed that cells treated with CH-AuNPs had enhanced fluorescence in comparison to the control, from 13% to 27% (Figure 5F), confirming O_2_^−^ production. Additionally, NAC inhibited O_2_^−^ production induced by CH-AuNP treatment. Finally, to determine the role of O_2_^−^ in cell death, we assessed phosphatidyl serine exposure with annexin V by flow cytometry in the presence of NAC. In Figure 5G, we show the detection of low fluorescence induced by CH-AuNPs (22%), and this fluorescence diminished in presence of NAC (6.5%). This indicated that low O_2_^−^ produced by CH-AuNPs in PBMCs stimulated with ConA were involved in the low cytotoxicity, and suggested that ROS are implicated in other metabolic processes.

### 3.6. CH-AuNPs Do Not Modify Cell Viability in Primary Lymphoid Cells during Proliferative Stimulus

**Figure 6:**
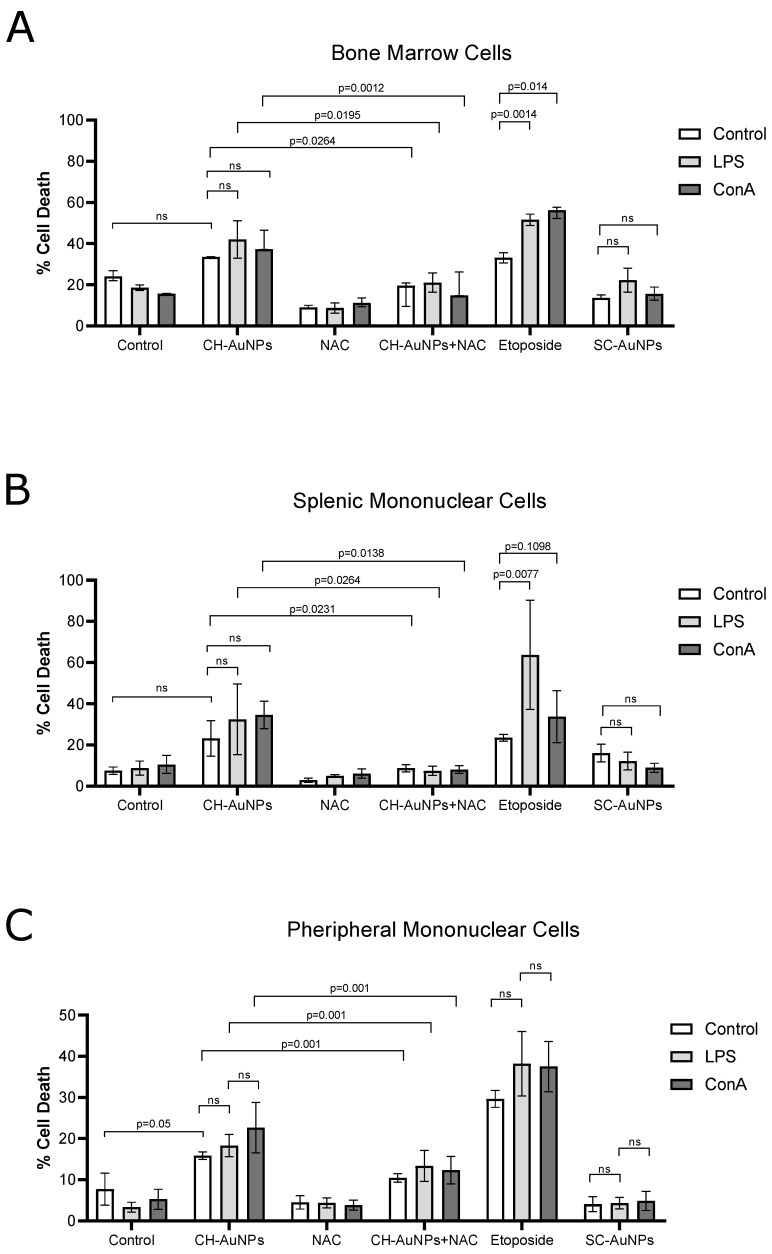
Cell-death analysis of CH-AuNPs, SC-AuNPs, and etoposide in BMCs, splenic mononuclear cells, and PBMCs under proliferative stimuli. Cell-death quantification of (**A**) BMC, (**B**) splenic mononuclear cells, and (**C**) PBMCs treated with CH-AuNPs (125 μM), SC-AuNPs (125 μM), and etoposide (100 mM) for 24 h, using lipopolysaccharide (LPS) and concanavalin A (ConA) for stimulation, and NAC as a ROS inhibitor. The results are presented as mean ± standard deviation of three different individuals. ns = not significant.

Proliferative cells are the principal target of chemotherapy, including cancer cells and non-cancer cells derived from the mouth, digestive system, hair follicles, and immune system. This is why one of the principal adverse effects of chemotherapy is the high cytotoxicity in immune-system cells. To determine the cytotoxicity of CH-AuNPs in immune system-derived cells, we tested cell death on splenic mononuclear cells, BMCs, and PBMCs, with or without the presence of two proliferative stimuli, lipopolysaccharide (LPS) and concanavalin A (ConA). We used etoposide, a widely used chemotherapeutic drug, and SC-AuNPs as controls. In Figure 6A, we can observe that CH-AuNPs and SC-AuNPs did not induce significant cell death in BM cells, which did not increase significantly during proliferative stimuli with LPS or ConA. In contrast, the cell death induced by etoposide in BM cells significantly increased under both proliferative stimuli (Figure 6A). In the splenic mononuclear cells analyses (Figure 6B), we can observe a similar pattern, in which CH-AuNPs and SC-AuNPs did not significatively decrease cell viability even in presence of proliferative stimuli. On the other hand, the cell death induced by etoposide significantly increased under LPS treatment. Finally, in the PBMC analysis (Figure 6C), the results showed that proliferative stimuli did not increase the cell death induced by CH-AuNPs or SC-AuNPs, contrary to etoposide, which was highly cytotoxic to PBMCs with or without the proliferative stimuli. Additionally, in the presence of NAC, the low cell death induced by CH-AuNPs decreased in BM cells (Figure 6A), splenic mononuclear cells (Figure 6B), and PBMCs (Figure 6C), indicating that ROS played a crucial role in cell death, even under proliferative stimuli of lymphoid cells.

## 4. Discussion

**Figure 7:**
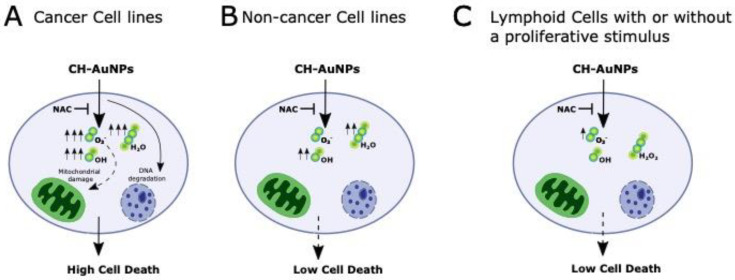
Schematic representation of CH-AuNPs’ effect on cancer and non-cancer cells. (**A**) In cancer cells, CH-AuNPs induced a loss of mitochondrial membrane potential and DNA degradation [17,18], and enhanced intracellular ROS production (O_2_^−^, •OH and H_2_O_2_). ROS were inhibited using NAC, which avoided cell death. (**B**) In non-cancer cell lines, CH-AuNPs enhanced •OH and H_2_O_2_ production (inhibited by NAC), leading to cell death. (**C**) In lymphoid cells, with or without proliferative stimulus, CH-AuNPs enhanced a slight O_2_^−^ production, which was inhibited by NAC.

We synthetized CH-AuNPs by a chemical method and obtained NPs with a surface plasmon resonance of 520 nm, a diameter of 3–10 nm, and a zeta potential (ZP) of +36.7 mV. These characteristics were similar to the ones previously reported for CH-AuNPs with cytotoxic properties in tumoral and leukemic cell lines [17,18]. CH-AuNPs did not decrease the cell viability of HUVECs, NIH3T3s and PBMCs more than 30% at the concentration at which 100% of cell-viability loss was observed in A549. SC-AuNPs and the synthesis precursors, chitosan and HAuCl_4_, did not exhibit cytotoxicity. The cell-death analysis confirmed that CH-AuNPs possessed potential cytotoxic activity against A549, Jurkat, L5178Y-R, and CEM cancer cell lines (Figure 7A), and lower toxicity to non-cancer (HUVEC and NIH3T3) cell lines (Figure 7B) and PBMCs (Figure 7C). This was similar to our previous reports on tumoral (HeLa and MCF-7) [17] and leukemic (K562 and CEM) cell lines [18].

The dispersity of NPs is involved in cytotoxicity, and may be related to the increase of cellular endocytosis and ROS [26]. Additionally, the interaction between cationic AuNPs and negatively charged plasma membrane were shown to be determinant for the cytotoxicity [27,28], and this positive charge of the CH-AuNPs could be also determine the selectivity to cancer cells. CH-AuNPs and SC-AuNPs showed similar polydispersity (0.3 for both); however, CH-AuNPs had a positive charge (+36.7 mV) compared to a negative charge for SC-AuNPs (−10 mV). Other cationic AuNPs showed similar cytotoxicity in a cervical cancer cell line (HeLa) and in a normal human dermal fibroblast cell line (NHDF) [27]. Physicochemical properties such as surface, size, and dispersity of NPs also determine their biological impact. Several shapes of AuNPs, such as rods, stars, and spheres, showed unselective cytotoxicity in osteosarcoma (143B, MG63) cell lines and in human fetal osteoblast (hFOB 1.19) [29]. Flower-shaped and spherical AuNPs synthesized with different precursors decreased cell viability in human endothelial cells [30,31] (HUVECs), which showed an intracellular accumulation of AuNPs [32]. Other authors revealed the attenuation of cell growth in different mammalian cell lines treated with AuNPs, including the NIH3T3 cell line [32,33]. AuNPs–calreticulin did not importantly affect the cell viability of HaCaT, HUVECs, and NIH3T3 cells [34]. AuNPs–PMAM showed effects in PBMCs [35], in contrast to green AuNPs obtained from *C. guianensis,* which showed antitumor activity without affecting PBMCs [36]. Other reports showed that antigen-presenting cells (APCs) effectively internalized chitosan-coated FAPLGA and SC-FA-PLGA nanoparticles, causing low cytotoxic effects [37]. AuNPs obtained from *Marsdenia tenacissima* by green synthesis [38], sodium citrate AuNPs [39], *Justicia adhatoda*–AuNPs [40], AuNPs synthetized using marine bacteria *Enterococcus* sp [40], and AuNPs in combination with irradiation [27] inhibited cell proliferation in a concentration-dependent manner and decreased cell viability in the A549 cell line. Other AuNPs (green synthesis) using *Illicium verum* showed cytotoxicity in the A549 cell line [41], and 4 nm AuNP induced cytotoxicity in vitro in the L5178Y cell line [42]. These data highlight the biological effects of AuNPs depending on the shape, size, and synthesis used, and remarkably, cell lineage.

CH-AuNPs induced H_2_O_2_ production in cancer cells and HUVECs, which was not observed in PBMCs or BM cells. In contrast, increased O_2_^−^ production induced by CH-AuNPs was observed in cancer and HUVEC cell lines, and in PBMCs. DCFDA is a fluorescein-based nonspecific and indirect probe that measures H_2_O_2_ and non-specifically detects other ROS, such as hydroxyl radicals (∙OH), peroxynitrite (ONOO^−^), and a heme protein [21]; however, none of these were detected in PBMCs. On the other hand, DHE is an intracellular ROS probe that is most commonly used for the detection of superoxide (O∙_2_^−^), although it also reacts with hydrogen peroxide (H_2_O_2_) in the presence of peroxidases, and with oxidases and cytochromes [22,43,44]. The O_2_^−^ produced by NAD(P)H oxidases, present in all cell types, participates in inflammation and may lead to toxic effects, and when produced at high levels, it may also modulate inflammation [45]. Here, we did not evaluate cytokine release, but it is possible that CH-AuNPs could induce a pro-inflammatory profile in PBMCs. Thus, further studies must be done to determine the effect of CH-AuNPs on the induction of other types of ROS, the mechanism leading to the specific redox modification in PBMCs, and their role in inflammation. Additionally, it would be important to determine if the differences in mitochondrial respiration and increased glucose consumption in cancer cells could lead to higher mitochondrial damage by CH-AuNPs, and thus ROS production in cancerous cells, rather than PBMCs, explaining these differences between normal and cancer cells.

The effect of anti-cancer agents on cell-cycle progression is important. Most, if not all, human cancer types show a deregulated control of G1 progression, a period in which cells decide whether to begin proliferation or stay quiescent [46]. In the cell-cycle analysis, we observed that CH-AuNPs did not induce cell-cycle alterations in HUVECs, similar to our observations in tumor (HeLa and MCF-7) [17] and leukemic (K562 and CEM) [18] cell lines. In PBMCs and BM cells, we did not observe a significant percentage of cells in the S and G2 phases, as these were primary cell cultures, in contrast to immortalized cell lines. We did not observe differences in either cell line during NAC treatment. Interestingly, NAC inhibited cell death induced by CH-AuNPs, which was lower in non-cancer cell lines. NAC is a precursor of L-cysteine and is a source of sulfhydryl groups in cells; it also interacts with ROS, making it a scavenger of free radicals such as •OH and H_2_O_2_ [47]. Because CH-AuNPs directly enhanced ROS production, pretreatment with NAC inhibited interaction with free radicals. Autophagy is a key protective mechanism against mitochondrial damage and the consequent ROS-induced cellular accumulation [48]. Previously, we observed pro-survival autophagy in leukemic cells [18]. It is probable that alterations in ROS production could increase autophagy on lymphoid cells to avoid cell death.

However, we previously tested the effect of CH-AuNPs on PBMCs, and proliferation was not previously induced in the cell model. Thus, we assessed the effect of CH-AuNPs on PBMCs in the presence of the mitogen ConA to induce proliferation in PBMCs. We observed that ConA induced DNA synthesis and cell division in PBMCs, as previously reported [25,49]. We did not observe alterations in cell viability or integrity in PBMCs during the proliferative state. In addition, we observed that CH-AuNPs enhanced O∙_2_^−^ production, similar to PBMCs alone and cancer cell lines, even if cytotoxicity was selective only to cancer cells. SC-AuNPs and AuNPs–PMAM increased intracellular ROS in the HepG2 cell line and in PBMCs, which mediated cytotoxicity [35]. Some studies revealed that SiO_2_ NPs induced oxidative stress and triggered a cytokine inflammatory response [50,51,52,53]. In addition, AuNPs capped with nucleic acid augmented PBMC proliferation in response to phytohemagglutinin, and increased release of IL-10 and IFN-γ in comparison to uncapped AuNPs [54] and IL-2 [55]. This suggested that ROS induced by CH-AuNPs could induce a proinflammatory response in PBMCs; these results reinforced that CH-AuNPs’ cytotoxicity is selective only to cancer cells, and is independent of the proliferative status. Previous reports observed that glyco-thiol AuNPs showed more cytotoxicity to the A549 cell line in comparison to PBMCs, because their hydrophobic nature allowed them to cross the cancerous cell membrane more easily [54]. The A549 cell line revealed selective internalization of S15-APT QDs via classical clathrin-dependent, receptor-mediated endocytosis, in comparison to normal human bronchial epithelial cells (BEAS2B) [56]. SC-PLGA NPs were internalized more efficiently than PLGA, presumably because of receptor-mediated endocytosis; among PBMCs, APCs showed higher uptake of both NP preparations than lymphocytes [37,57]. This highlighted the different effects of CH-AuNPs on healthy and cancerous cells, which could also be due to molecular differences and the different receptors panel in cancer cells.

Finally, to test the cytotoxicity of CH-AuNPs in other immune-system cells in proliferation, we tested cell death in BMCs, splenic mononuclear cells, and PBMCs with or without the presence of two different proliferative stimuli that mimic infectious diseases (LPS and ConA) and treated with CH-AuNPs. Our results showed that CH-AuNPs and SC-AuNPs did not increase their cytotoxicity in immune-system cells, even in presence of a proliferative stimulus (Figure 7), in contrast to the conventional chemotherapy etoposide. Our data showed similar results to other evidence, in which two chemotherapies, etoposide and campotothecin, demonstrated the ability to induce apoptosis in proliferative-peripheral lymphocytes [58]. In addition, another study showed that cisplatin and gemcitabine inhibited PBMC proliferation induced by PHA [59].

## 5. Conclusions

Taken together, our results highlighted the selectivity of CH-AuNPs to cancer cells in a ROS-dependent manner (Figure 7), discarding a consistent cytotoxicity upon proliferative cells, including endothelial, fibroblast, and lymphoid cells, and suggested their application in cancer treatments without affecting immune cells. Differences were found when detecting ROS production, as we were unable to detect ROS production in PBMCs when using DCFDA, but we detected them using DHE, while in all cell lines, ROS were detected irrespective of the detection method. In addition, we did not observe significant cell death in lymphoid cells using proliferative stimuli that mimicked infection. This work opens the door to further research to determine the specific mechanisms for ROS production induced by CH-AuNPs in PBMCs, as well as in vivo experiments exploiting their selectivity to cancer cells irrespective of the proliferative status of lymphoid cells.

## Data Availability

The data presented in this study are available on request from the corresponding author.
